# Novel *SETBP1* mutation in a chinese family with intellectual disability

**DOI:** 10.1186/s12920-023-01649-x

**Published:** 2023-10-05

**Authors:** Le Wang, Xu-Dong Wang, Bo Yang, Xue-Meng Wang, Yu-Qian Peng, Hang-Jing Tan, Hong-Mei Xiao

**Affiliations:** 1 School of Basic Medical Science, Hunan University of Medicine, Huaihua, Hunan China; 2 Center for System Biology, Data Sciences, and Reproductive Health, School of Basic Medical Science, Central South University, Changsha, Hunan China

**Keywords:** SETBP1, Intellectual disability, Autosomal dominant mental retardation 29, Whole-genome sequencing

## Abstract

**Background:**

Intellectual disability (ID) is characterized by an IQ < 70, which implies below-average intellectual function and a lack of skills necessary for daily living. ID may occur due to multiple causes, such as metabolic, infectious, and chromosomal causes. ID affects approximately 1–3% of the population; however, the cause can be identified in only 25% of clinical patients.

**Methods:**

To find the cause of genetic ID in a family, we performed whole-exome sequencing and Sanger sequencing to confirm the presence of a *SETBP1* variant and real-time quantitative polymerase chain reaction to detect *SETBP1* expression in the proband and normal controls.

**Results:**

A novel variant, c.942_943insGT (p. Asp316TrpfsTer28), was found in *SETBP1*. Furthermore, we observed that *SETBP1* expression in patients was only 20% that of normal controls (P < 0.05).

**Conclusion:**

A heterozygous variant in *SETBP1* associated with ID was found. This report provides further evidence for its genetic basis and support for clinical genetic diagnosis.

**Supplementary Information:**

The online version contains supplementary material available at 10.1186/s12920-023-01649-x.

## Background

Intellectual disability (ID) is characterized by an IQ < 70, which may occur due to environmental or genetic causes. Environmental impacts always occur during pregnancy and childbirth. Toxic substances, pathogens, drugs, and injuries can cause ID. One of the most common mechanisms is through the toxic effects of alcohol, which presents as fetal alcohol syndrome. Genetic abnormalities may cause ID by inducing neurodevelopmental defects or neurodegeneration [1]. Several ID-related genes have been identified, such as *SETBP1* [5], *HGPRT* [2], *FMR1* [3], and *MECP2* [4]. Variants of *SETBP1* can cause mental retardation, autosomal dominant 29 (MRD29), also known as SETBP1 disorder, which is a condition that involves mental retardation, speech and language problems, and non-specific facial features [5, 6]. Little is known about the function of the SETBP1 protein except that it can bind with the SET protein in a particular domain [7].

Here, we report three patients from a non-consanguineous family with the same variant in *SETBP1* (c.942_943insGT, p.Asp316TrpfsTer28). They exhibited similar syndromes, including mental retardation and speech problems. The heterozygous frameshift variant led to the truncation of the SETBP1 protein. We suspect that this variant was the cause of ID in this family.

## Methods

### Patient characteristics

The proband was a 27-year-old woman. She was the third child of non-consanguineous parents. She had difficulty communicating and was unable to go out by herself. Her 29-year-old sister had the same symptoms, while her 31-year-old sister was healthy and had normal cognition.

**Fig. 1:**
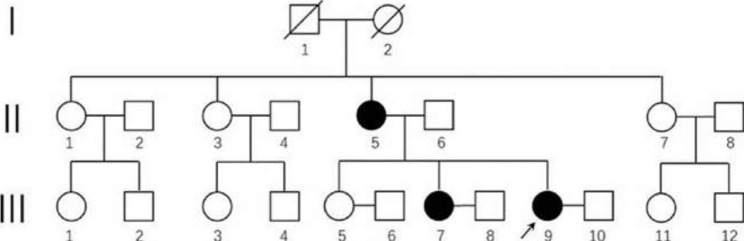
Clinical features. Genogram. The proband (III-9) is indicated by the arrow

As Fig. 1 shows, the proband’s mother (II-5), sister (III-7), and her sister’s daughter (IV-2) presented the same symptoms. We collected peripheral blood samples from the proband, her mother, and her unaffected elder sister (III-5) and extracted genomic DNA (gDNA). The three individuals provided written approval for their participation in this study and its publication. This study was approved by the Ethics Committee of Central South University, China, and was performed in accordance with the principles of the Helsinki Declaration II.

### Exome sequencing

Sequencing and analysis were performed as previously described [8]. Samples were submitted to BGI Genomics (Shenzhen, China), which used liquid-capture systems to efficiently capture and enrich human DNA across the entire exome and then provided BGISEQ-500 and HiSeq (Illumina, San Diego, CA) platform services. First, single-stranded circular DNA was replicated through rolling circles to produce DNA nanoballs (DNBs). High-density DNA nanochip technology was used to fix the obtained DNBs on an arrayed silicon chip. A 100-bp paired-end sequence was obtained by combined probe anchor polymerization and double-end sequencing with multiple displacement amplification. Information analysis was initiated with the raw sequencing data. The raw data were filtered to remove adapters, low-quality bases, and undetected bases (represented by N) and compared to the reference genome for single nucleotide polymorphism detection and indel or copy number variation analysis. We then screened harmful sites or genes related to ID according to mutation harmfulness, sample condition, and gene functional phenotype. Thereafter, we used Sanger sequencing to confirm that all patients in this family shared the same *SETBP1* variant. Specific PCR primers (SETBP1-F: 5-CACATGGACTGGTCCACCAAC-3 and SETBP1-R: 5-TTTTACTGGACTTTTTCTTGCTGC-3) were used to amplify the target region.

### Real-time quantitative polymerase chain reaction

Total RNA of the proband and healthy controls was isolated from peripheral blood using TRIzol (Invitrogen, Carlsbad, CA). Using the GoScript Reverse Transcription System kit (Promega, Madison, WI), we subjected RNA to reverse transcription. The relative mRNA levels of *SETBP1* were detected by using real-time quantitative polymerase chain reaction (RT-PCR). Total RNA was converted to cDNA after 40 amplification cycles as follows: 95 °C for 300 s, 95 °C for 10 s, 58 °C for 10 s, and 72 °C for 30 s. The CFX96 Touch Real-Time PCR Detection System (Bio-Rad Laboratories, Hercules, CA) and MonAmp ChemoHS qPCR Mix (Monad Biotech, Zhuhai, China) were used. *GAPDH* was used for standardization. The RT-PCR primers are shown in Supplementary Material Table 1.

### Statistical analysis

To guarantee the reproducibility of our results, each experiment was performed at least three times, and representative results are shown as mean ± standard deviation. Using the Kruskal‒Wallis test with Steel’s post hoc test, we evaluated differences between the control and proband groups. A difference was regarded as statistically significant at a P value < 0.05.

## Results

### Case Presentation

The proband (III-9) was a 27-year-old woman with moderate ID. The proband’s mother (II-5), sister (III-7), and her sister’s daughter (IV-2) presented the same symptoms. The proband’s mother (II-5) underwent brain magnetic resonance imaging with normal results. In addition, following primary genetic tests, including the chromosome G-binding test and chromosome microarray analysis (Affymetrix CytoScan 750 K Array) test, the proband did not receive an etiological diagnosis. The clinical information of the patients is presented in the Supplementary Material Table 2.

### Gene Identification

**Fig. 2:**
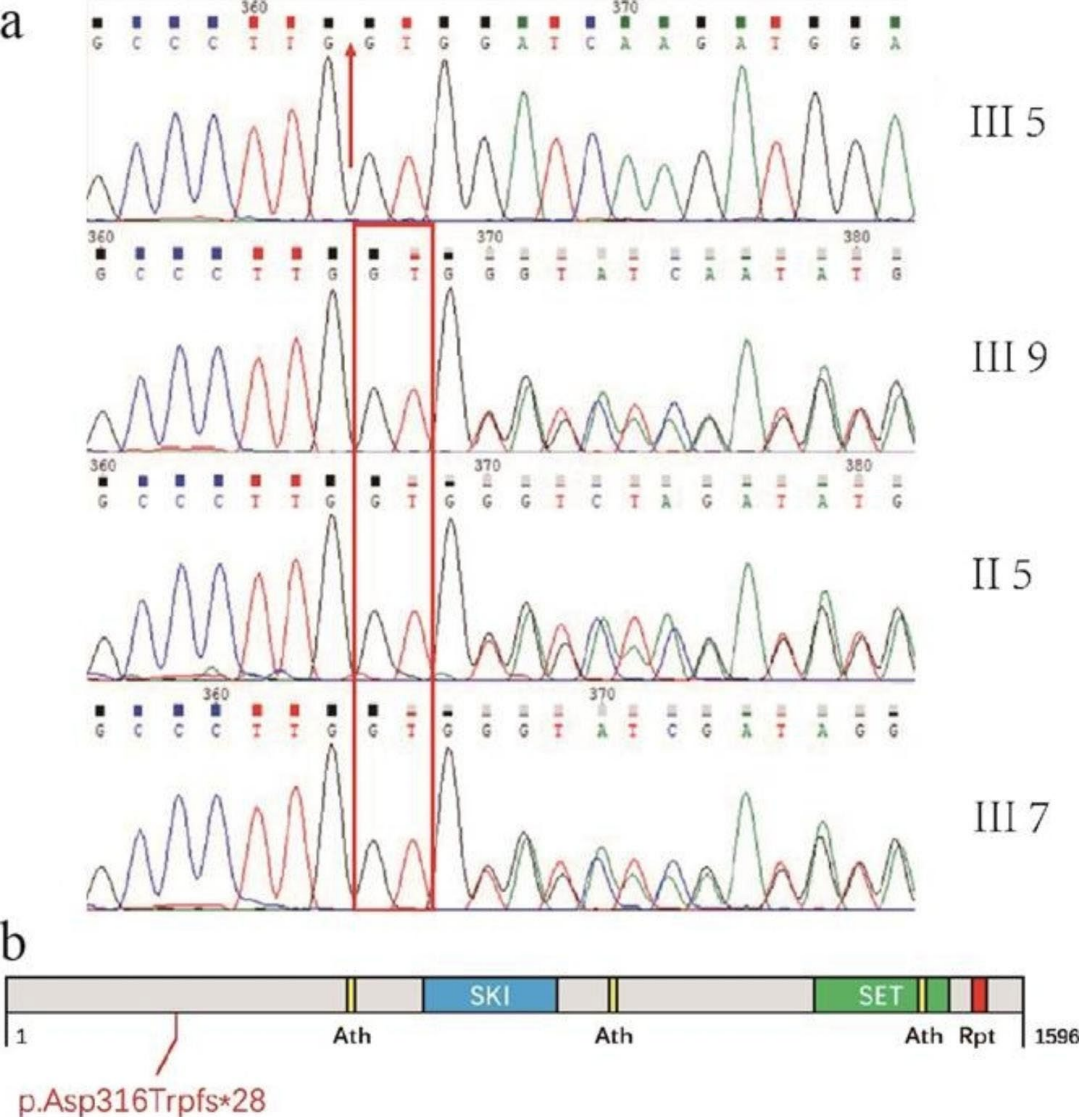
Results of Sanger sequencing. The healthy sister (III-5) had a normal gene sequence. The proband (III-9), proband’s sister (III-7), and proband’s mother (II-5) had the same variant (c.942_943insGT, p. Asp316TrpfsTer28) in *SETBP1*

We obtained DNA samples from the peripheral blood samples of II-5, III-5, III-9, and III-7 and analyzed these DNA samples through whole-exome sequencing. We found 15 known pathogenic genes that might cause ID.We searched for research reports on the pathogenicity of these genes and found that the phenotype of the patients matched that of MRD29. MRD29, also known as SETBP1 disorder, which is a disease characterized by ID, speech and language problems, and non-specific facial features. In the reported of MRD29, 94% (77/82) of patients had intellectual disabilities, language and motor developmental delays, 47% (37/78) had visual impairments, 53% (17/32) had hearing impairments, and 56% (46/82) had attention/attention deficit issues. A few patients also exhibited other phenotypes (Supplementary Material Table 3). Therefore, we performed Sanger sequencing to validate the *SETBP1* mutation in the affected individuals in the pedigree (Fig. 2). Based on the phenotype and variant type of the patients, we identified a *SETBP1* variant (c.942_943insGT, p. Asp316TrpfsTer28) as the causative pathogenic variant.

### *SETBP1* expression

**Fig. 3:**
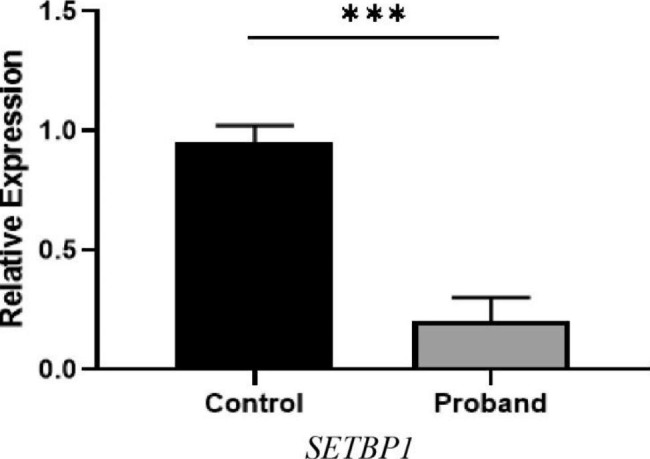
*SETBP1* mRNA expression. *SETBP1* mRNA expression in probands was significantly lower than that in controls (P < 0.05)

To verify the decreased *SETBP1* expression in the proband, we extracted mRNA from the proband’s peripheral blood and healthy controls for RT-PCR analysis. The results showed that *SETBP1* mRNA levels in the proband were 20% those of her normal sister (III-5) (Fig. 3), indicating that this variant led to a decrease in *SETBP1* expression and caused ID.

## Discussion

Here, we describe a frameshift variant in *SETBP1* in a family with ID, which caused a decrease in *SETBP1* expression.

Variants in SETBP1 lead to MRD29 and Schinzel Giedion syndrome (SGS) [5–7]. MRD29 is characterized by mild or moderate mental retardation and growth retardation, with most reported variants being frameshift and nonsense variants in *SETBP1*, while SGS is characterized by growth retardation, special facial features, and multiple deformities, among others, and most reported variants are missense variants in *SETBP1* with a typical variant hotspot in the fourth exon. At present, it is suggested that patients with frameshift and nonsense variants in *SETBP1* have haploinsufficiency and an MRD29 phenotype, while missense heterozygous variants in hotspots in *SETBP1* lead to dominant-negative or gain-of-function effects, and patients develop an SGS phenotype [8–10]. Due to the phenotypic heterogeneity of MRD29, there are currently no unified diagnostic criteria. We found that mild intellectual disability, language delay, and delayed motor development were common features among the reported 82 cases. Therefore, by combining whole-exome sequencing to detect *SETBP1* mutation types, a more effective diagnosis of MRD29 patients can be made, enabling timely treatment interventions.

SETBP1 is a binding protein that acts as a protein phosphatase 2 A (PP2A) inhibitor by forming a dimer with a SET protein [11, 12]. Ephraim et al. reported that PP2A can inhibit nerve growth in nerve cells. During development, SET-PP2A regulates the differentiation and proliferation of nerve cells, while overexpression or knockout of SET proteins in rat hippocampal neurons can suppress neurites and axon elongation [13]. Therefore, the decreased *SETBP1* expression due to the frameshift variant leads to instability of the SETBP1/SET dimer, which cannot inhibit the function of PP2A, thus affecting the development of nerve cells. We consider this a potential molecular mechanism explaining the link between the *SETBP1* frameshift variant and mental retardation.

In conclusion, in this family, we found a truncated variant in *SETBP1* leading to MRD29. Owing to haploinsufficiency, the patients’ symptoms only included moderate retardation and language delay; for this type of MRD29 patient, whole-exome sequencing is more accurate for diagnosis and can also be helpful for clinical treatment.

### Electronic supplementary material

Below is the link to the electronic supplementary material.

Supplementary Material 1

## Data Availability

The datasets generated for this study can be found in the NCBI Sequence Read Archive (SRA) database (accession number: PRJNA993619), (direct link: https://www.ncbi.nlm.nih.gov/sra/PRJNA993619). Individual-level data can be requested from the corresponding author (*hmxiao@csu.edu.cn*).

## Abbreviations

DNB: DNA nanoball
ID: Intellectual disability
MRD29: Mental retardation, autosomal dominant 29
RT-PCR: real-time quantitative polymerase chain reaction
SGS: Schinzel Giedion syndrome
